# ABCA1 deficiency contributes to podocyte pyroptosis priming via the APE1/IRF1 axis in diabetic kidney disease

**DOI:** 10.1038/s41598-023-35499-5

**Published:** 2023-06-14

**Authors:** Marie Ito, Gloria Michelle Ducasa, Judith David Molina, Javier Varona Santos, Shamroop Kumar Mallela, Jin Ju Kim, Mengyuan Ge, Alla Mitrofanova, Alexis Sloan, Sandra Merscher, Imari Mimura, Alessia Fornoni

**Affiliations:** 1 Department of Medicine, Katz Family Division of Nephrology and Hypertension, Peggy and Harold Katz Family Drug Discovery Center, University of Miami, Miller School of Medicine, University of Miami, Miami, FL 33136 USA; 2 Division of Nephrology and Endocrinology, The University of Tokyo Graduate School of Medicine, Tokyo, Japan

**Keywords:** Biochemistry, Cell biology, Endocrinology, Nephrology

## Abstract

Decreased ATP Binding Cassette Transporter A1 (ABCA1) expression and caspase-4-mediated noncanonical inflammasome contribution have been described in podocytes in diabetic kidney disease (DKD). To investigate a link between these pathways, we evaluated pyroptosis-related mediators in human podocytes with stable knockdown of ABCA1 (siABCA1) and found that mRNA levels of IRF1, caspase-4, GSDMD, caspase-1 and IL1β were significantly increased in siABCA1 compared to control podocytes and that protein levels of caspase-4, GSDMD and IL1β were equally increased. IRF1 knockdown in siABCA1 podocytes prevented increases in caspase-4, GSDMD and IL1β. Whereas TLR4 inhibition did not decrease mRNA levels of IRF1 and caspase-4, APE1 protein expression increased in siABCA1 podocytes and an APE1 redox inhibitor abrogated siABCA1-induced expression of IRF1 and caspase-4. RELA knockdown also offset the pyroptosis priming, but ChIP did not demonstrate increased binding of NFκB to IRF1 promoter in siABCA1 podocytes. Finally, the APE1/IRF1/Casp1 axis was investigated in vivo. APE1 IF staining and mRNA levels of IRF1 and caspase 11 were increased in glomeruli of BTBR ob/ob compared to wildtype. In conclusion, ABCA1 deficiency in podocytes caused APE1 accumulation, which reduces transcription factors to increase the expression of IRF1 and IRF1 target inflammasome-related genes, leading to pyroptosispriming.

## Introduction

Diabetic kidney disease (DKD) is the one of the main causes of end-stage kidney disease and among the top ten causes of death globally^1^. It also constitutes an independent risk factor of mortality and cardiovascular events in patients with diabetes^2^. Podocytes, which are terminally differentiated epithelial cells with low ability of self-renewal or proliferation, are key constituents of the glomerular filtration barrier. Clinical and experimental data suggest that podocyte loss correlates with the development of albuminuria, indicating the importance of podocyte injury in DKD^3,4^.

We previously demonstrated decreased ATP-binding cassette A1 (ABCA1) expression in glomerular transcripts from patients with DKD and in human podocytes exposed to sera from patients with DKD when compared to patients with diabetes and without DKD. Podocyte-specific Abca1 KO mice were sensitized to DKD injury, and both genetic and pharmacological induction of ABCA1 ameliorated DKD^5,6^. While the primary function of ABCA1 has been largely recognized as a cholesterol/phospholipid exporter, it has become clear that ABCA1 also exports reduction–oxidation factor 1 (Ref-1)/Apurinic/apyrimidinic endonuclease 1 (APE1), a transcription factor reducer and AP endonuclease^7^.

Pyroptosis, a form of inflammatory cell death, was found to contribute to podocyte loss in DKD with the main focus on canonical pyroptosis^8^. Pyroptosis requires two steps: priming and activation of the inflammasome. The priming step, in which the inflammasome components are transcriptionally upregulated, allows for the subsequent activation of inflammasome. In canonical pyroptosis, this transcriptional upregulation is induced through the binding of pathogen- and damage-associated molecular patterns (PAMPs and DAMPs) to pattern recognition receptors (PRRs) such as Toll-like receptors (TLRs) or through cytokines such as tumor necrosis factor (TNF) and IL1β, thus leading to NFκB activation^9^. Non-canonical pyroptosis is initiated by caspase-4/5 in human or caspase-11 in mice, and eventually causes downstream canonical inflammasome formation^10^. Non-canonical pyroptosis was recently found to mediate podocyte injury in DKD^11^. The main inducers of non-canonical pyroptosis known today are lipopolysaccharide (LPS) and activation of TLR4 signaling^9^. Indeed, activation of TLR4 signaling was shown to enhance pyroptosis in tubular cells in experimental DKD^12^. However, the mechanisms leading to non-canonical pyroptosis priming in podocytes remain unclear.

In this study, we demonstrate that ABCA1 deficiency in DKD contributes to the increase in non-canonical pyroptosis-related genes such as caspase-4/11, gasdermin D, caspase-1 and IL1β but not NLRP3. We also show that ABCA1 deficiency-induced priming of non-canonical pyroptosis is mediated by accumulated APE1, subsequent activation of transcription factors via reduction by APE1 and the enhancement of IRF1 transcription. Therefore, we propose the ABCA1/APE1/IRF1 axis to be a novel inducer of non-canonical pyroptosis in DKD.

## Results

### Non-canonical pyroptosis-related genes are upregulated in ABCA1 KD podocytes

**Figure 1:**
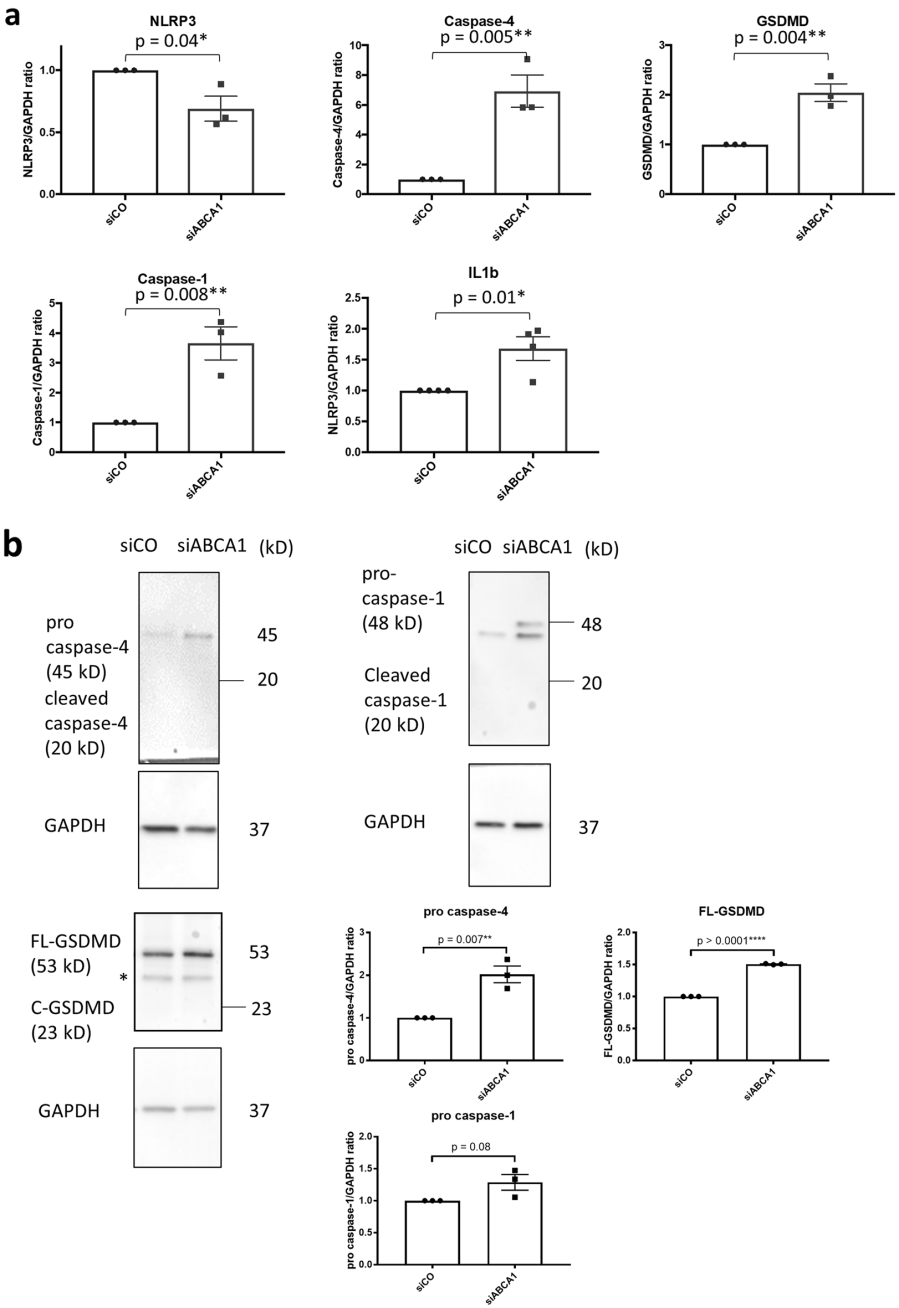

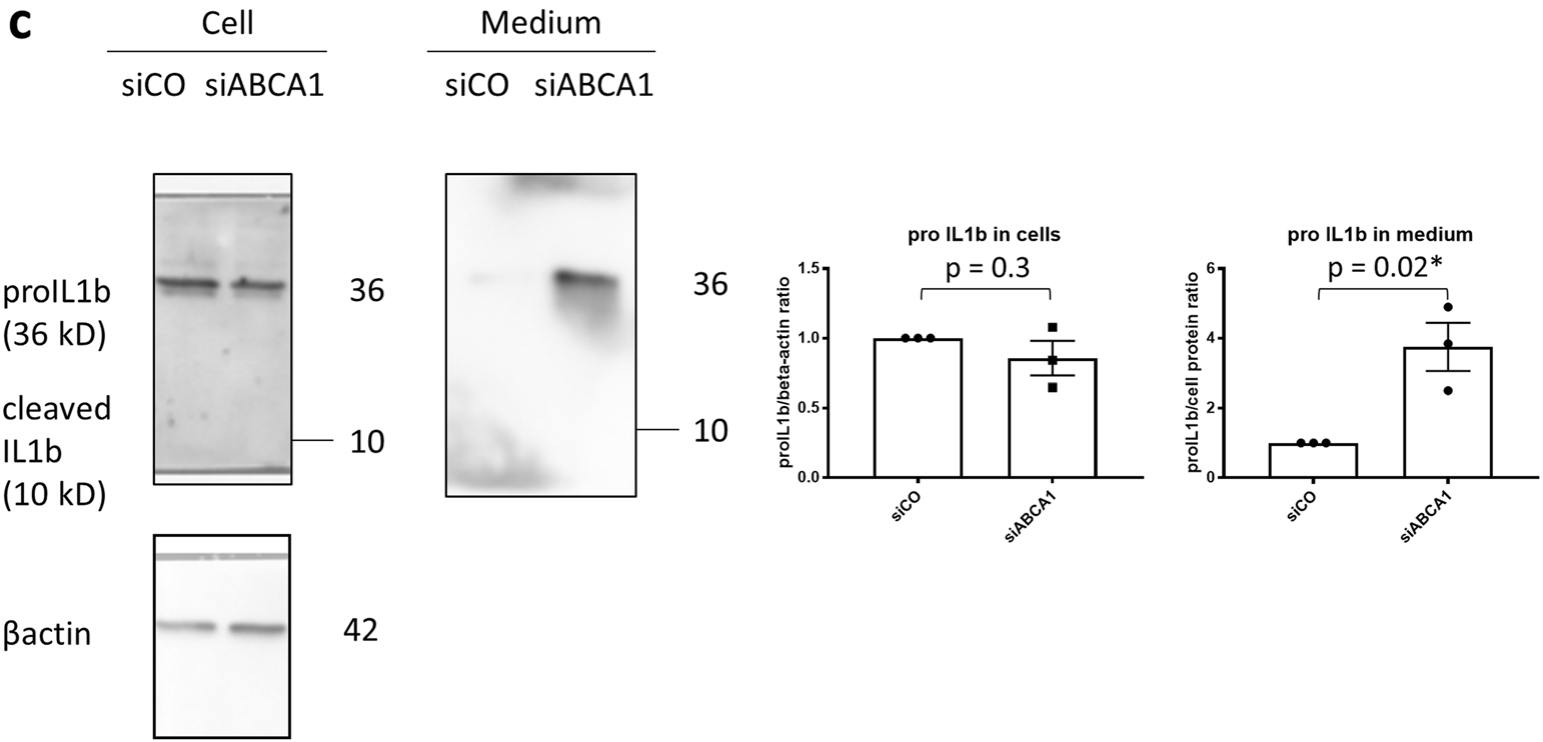
mRNA expression and uncleaved protein levels of non-canonical pyroptosis-related genes are increased in siABCA1 podocytes. (**a**) mRNA levels of non-canonical pyroptosis-related genes in siCO and siABCA1 podocytes. (**b**) Protein levels of uncleaevd or pro- form of non-canonical pyroptosis-related genes in siCO and siABCA1 podocytes. The bands indicated with *(around 35 kD) in the FL-GSDMD blot are nonspecific bands. The uncropped blots are in the Supplementary Fig. S1a–c. (**c**) Protein levels of pro-IL1b in cell lysates and supernatants of siCO and siABCA1 podocytes. N = 3 for each group except for IL1β mRNA, which is N = 4. The uncropped blots are in the Supplementary Fig. S1a,d.

We previously described that decreased ABCA1 expression in podocytes precedes podocyte injury in DKD and sensitizes podocytes to it^5,6^. Pyroptosis was recently identified as a novel mechanism of podocyte injury in DKD. However, the link between ABCA1 deficiency and pyroptosis has not been investigated^8^. To examine whether ABCA1 deficiency contributes to pyroptosis in podocytes, we measured mRNA levels of genes involved in pyroptosis in ABCA1 knockdown podocytes (siABCA1). mRNA expression of caspase-4, GSDMD, caspase-1 and IL1β was significantly increased in siABCA1 podocytes compared to control (p = 0.005 for caspase-4, 0.004 for GSDMD, 0.008 for caspase-1 and 0.01 for IL1β), whereas NLR family pyrin domain-containing protein 3 (NLRP3) expression decreased (Fig. 1a), in contrast to the previous reports indicating that NLRP3 expression is increased in podocytes treated with high glucose and in experimental DKD^8,13^. At the protein level, caspase-4 and GSDMD were significantly increased in siABCA1 compared to siCO podocytes (p = 0.007 for pro caspase-4 and 0.0001 for FL-GSDMD), while caspase-1 expression was increased but at a level that did not reach statistical significance (p = 0.08) (Fig. 1b). Although IL1β protein in cell lysates did not increase in siABCA1, we found increased IL1β precursor in the supernatants of siABCA1 podocytes when compared to siCO (Fig. 1c). Caspase-4 and GSDMD also increased and caspase-1 had the tendency to increase in their non-cleaved inactive forms in siABCA1 podocytes (Fig. 1a,b). These data demonstrated that ABCA1 knockdown in podocytes primes cells for non-canonical pyroptosis but is not sufficient to activate pyroptosis, which is consistent with our previous observation that ABCA1 deficiency contributes to DKD progression but is not sufficient to cause podocyte injury by itself^6^.

### Non-canonical pyroptosis activation induced by LPS electroporation is enhanced in ABCA1 KD podocytes

**Figure 2:**
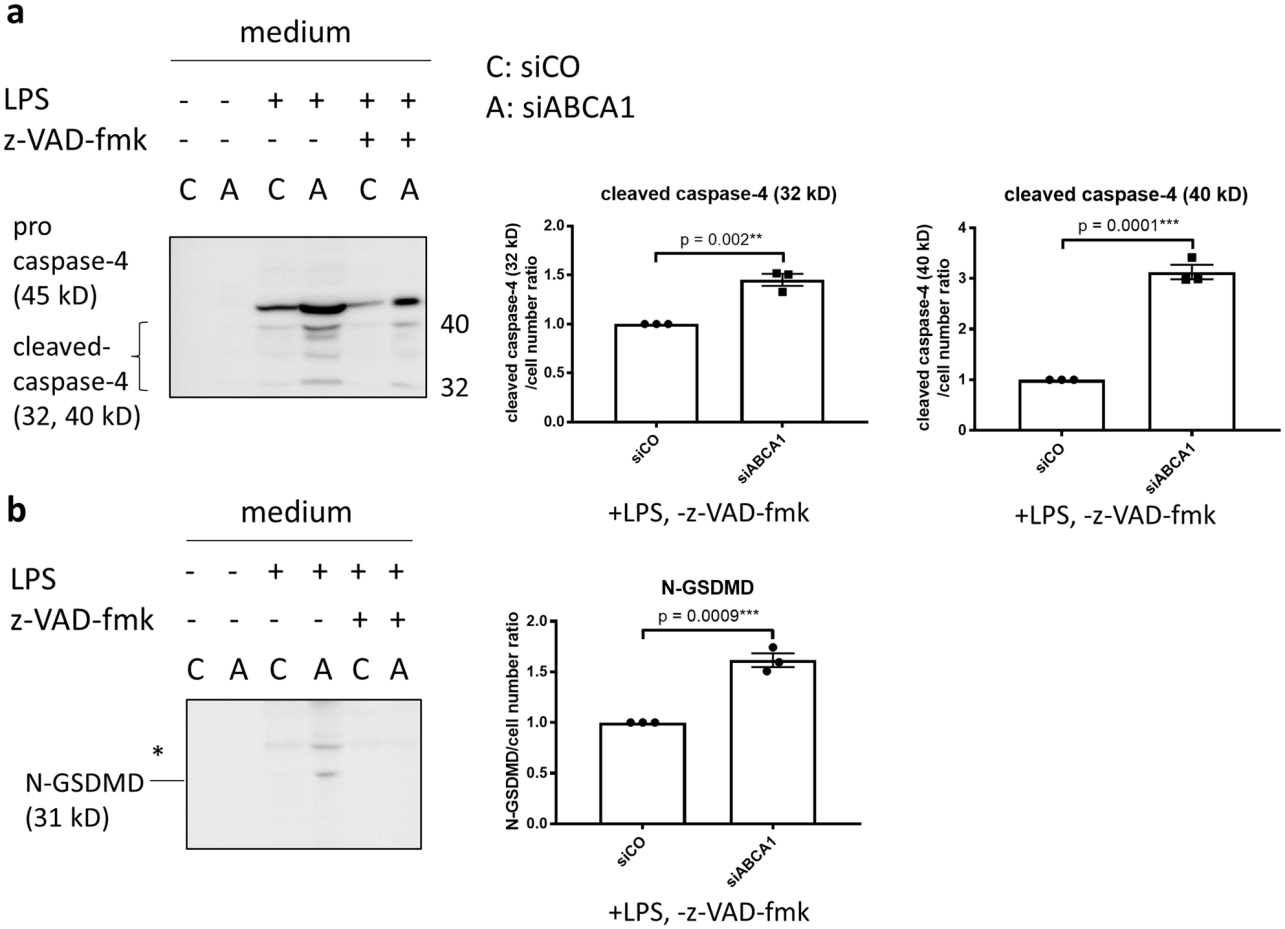
Protein levels of cleaved caspase-4 and N-GSDMD activated by LPS electroporation are increased in siABCA1 podocytes. (**a**) Protein levels of cleaved caspase-4 in siCO and siABCA1 podocytes. (**b**) Protein levels of N-GSDMD in siCO and siABCA1 podocytes. The uncropped blots are in the Supplementary Fig. S2a,b. N = 3 for each group. Each lane contains medium from the same cell number.

Next, we investigated whether the upregulation of pyroptosis-related genes in ABCA1 KD podocytes contributes to non-canonical pyroptosis activation. Caspase-4 and GSDMD is cleaved and excreted into medium by LPS electroporation, which is compatible with previous reports demonstrating non-canonical pyroptosis activation by LPS electroporation^14,15^. Pan-caspase inhibitor zVAD-fmk reverses the cleavage (Fig. 2a,b). ABCA1 KD significantly enhanced the cleavage (p = 0.002 for cleaved caspase-4 (32 kD), 0.0001 for cleaved caspase-4 (40 kD) and 0.0009 for N terminal-GSDMD (N-GSDMD)).

### IRF1 orchestrates the upregulation of non-canonical pyroptosis-related genes in ABCA1 KD podocytes

**Figure 3:**
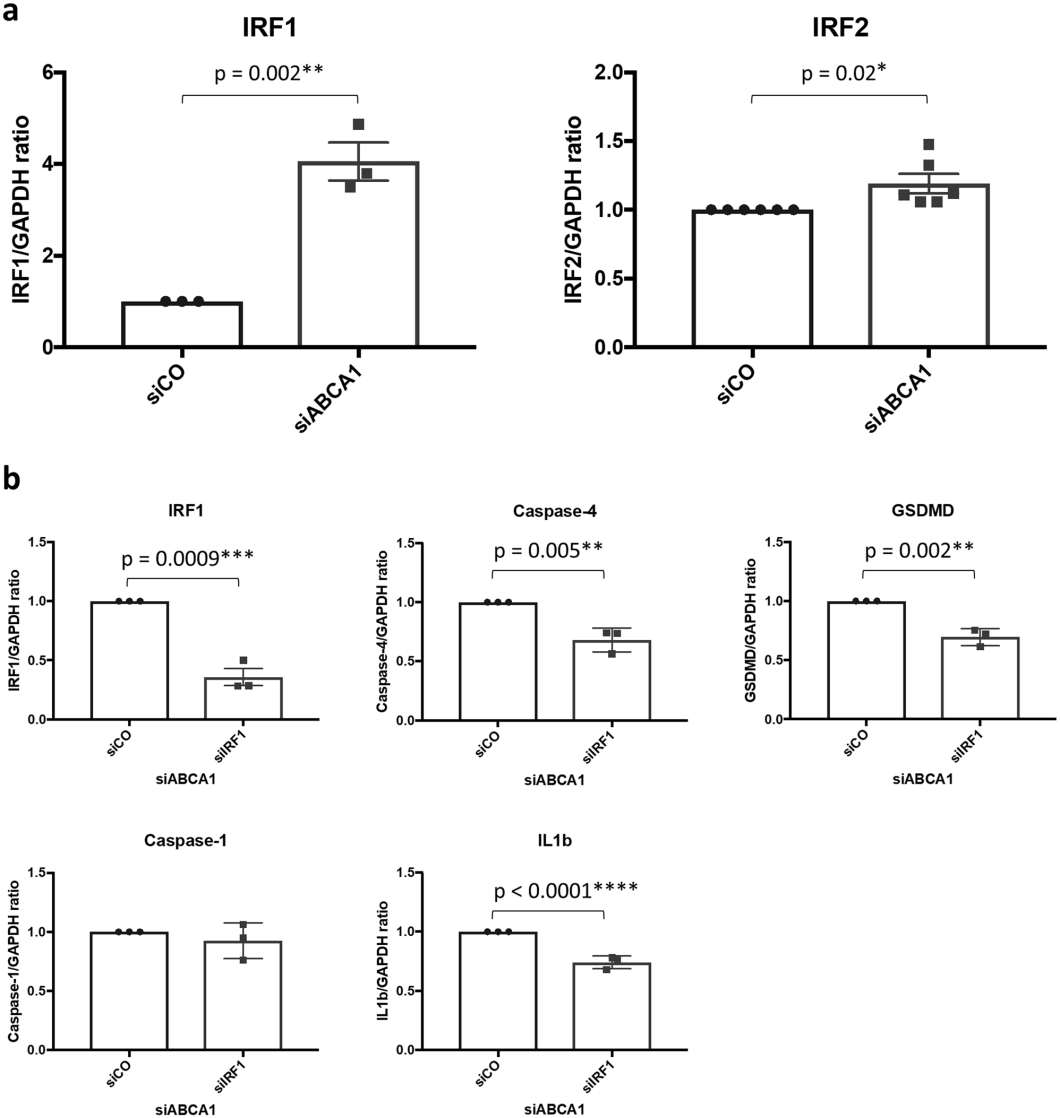
IRF1 regulates non-canonical pyroptosis-related genes in siABCA podocytes. (**a**) mRNA levels of IRFs in siCO and siABCA1 podocytes. (**b**) mRNA levels of non-canonical pyroptosis-related genes in siABCA1 podocytes transiently transfected with siCO and siIRF1. N = 3 for each group except for IRF2, which is N = 6.

Interferon regulatory factor 1 and 2 (IRF1 and 2) are the transcription factors which regulate non-canonical pyroptosis with cell-type specific target genes. For example, in human U937 cells, IRF2 regulates caspase-4 and -1 but not GSDMD and in human EA.hy926 hybrid endothelial cells IRF2 regulates GSDMD but not caspase-4 nor -1^16,17^. IRF1 compensates for IRF2 deficiency^18^. To explore whether IRF1 or 2 plays a role in regulating non-canonical pyroptosis-related genes in siABCA1 podocytes, we conducted qPCR for IRF1 and 2 gene expression in siABCA1 podocytes. Whereas the expression of IRF2 augmented significantly with larger n but to a small extent in siABCA1 podocytes (siABCA1/siCO ratio: 1.191 ± 0.0699, p = 0.02, N = 6), that of IRF1 were substantially increased (siABCA1/siCO ratio: 3.052 ± 0.4136, p = 0.002, N = 3) (Fig. 3a). In order to establish if IRF1 mediates the association between ABCA1 deficiency and pyroptosis, we next investigated whether siRNA knockdown for IRF1 in siABCA1 podocytes would offset the ABCA1 knockdown-induced changes in gene expression. We found mRNA levels of caspase-4, GSDMD and IL1β were significantly decreased in siIRF1 in siABCA1 podocytes (p = 0.005 for caspase-4, 0.002 for GSDMD, < 0.0001 for IL1β) while those of caspase-1 were not affected (Fig. 3b). Thus, we concluded that increased IRF1 is responsible for the upregulation of caspase-4, GSDMD and IL1β in siABCA1 podocytes.

### TLR4 does not mediate IRF1 upregulation in ABCA1 KD podocytes

**Figure 4:**
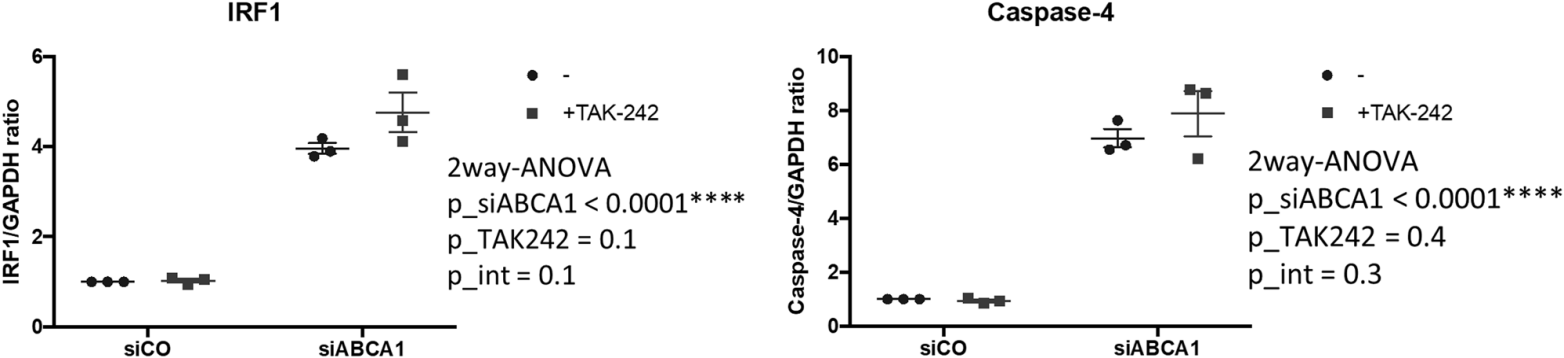
TLR4 is not involved in the regulation of IRF1 or caspase-4. mRNA levels of IRF1 and caspase-4 in siCO and siABCA1 cells treated with TAK-242 (TLR4 inhibitor). N = 3 for each group.

TLR4 signaling contributes to non-canonical pyroptosis priming^9^. ABCA1 deficiency in macrophages was found to enhance TLRs signaling by increasing TLRs (including TLR4) trafficking to plasma membrane lipid rafts^12,19^. To explore whether TLR4 signaling contributes to IRF1 and its downstream gene expression changes we observed in siABCA1 podocytes, we treated podocytes with TAK-242, a TLR4 inhibitor. The mRNA levels of IRF1 and caspase-4 were not significantly influenced by the inhibition of TLR4 (Fig. 4). These data suggest that TLR4 signaling does not contribute to ABCA1 deficiency-induced non-canonical pyroptosis priming in podocytes in our system.

### Reducing ability of APE1 mediates IRF1 upregulation in ABCA1 KD podocytes

**Figure 5:**
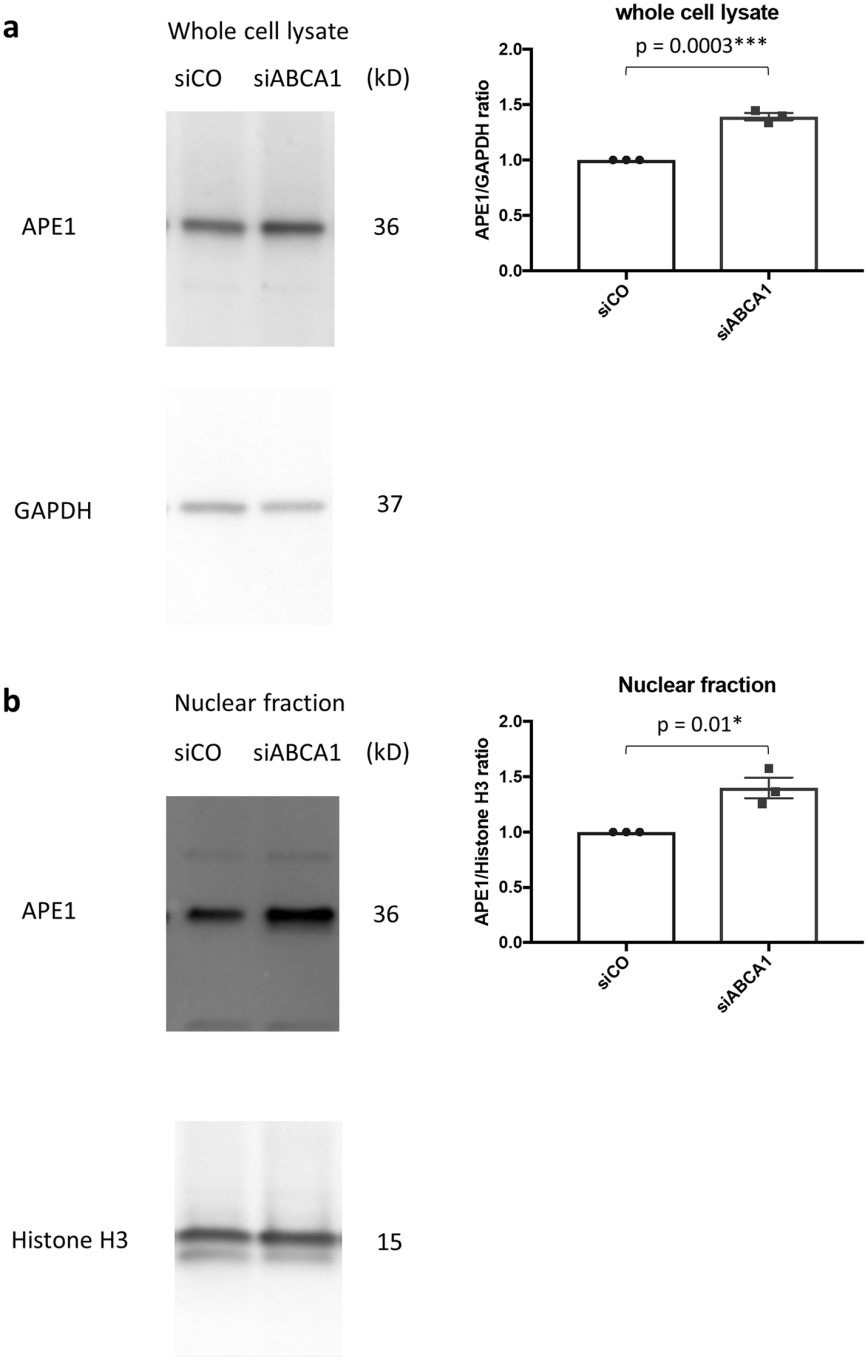

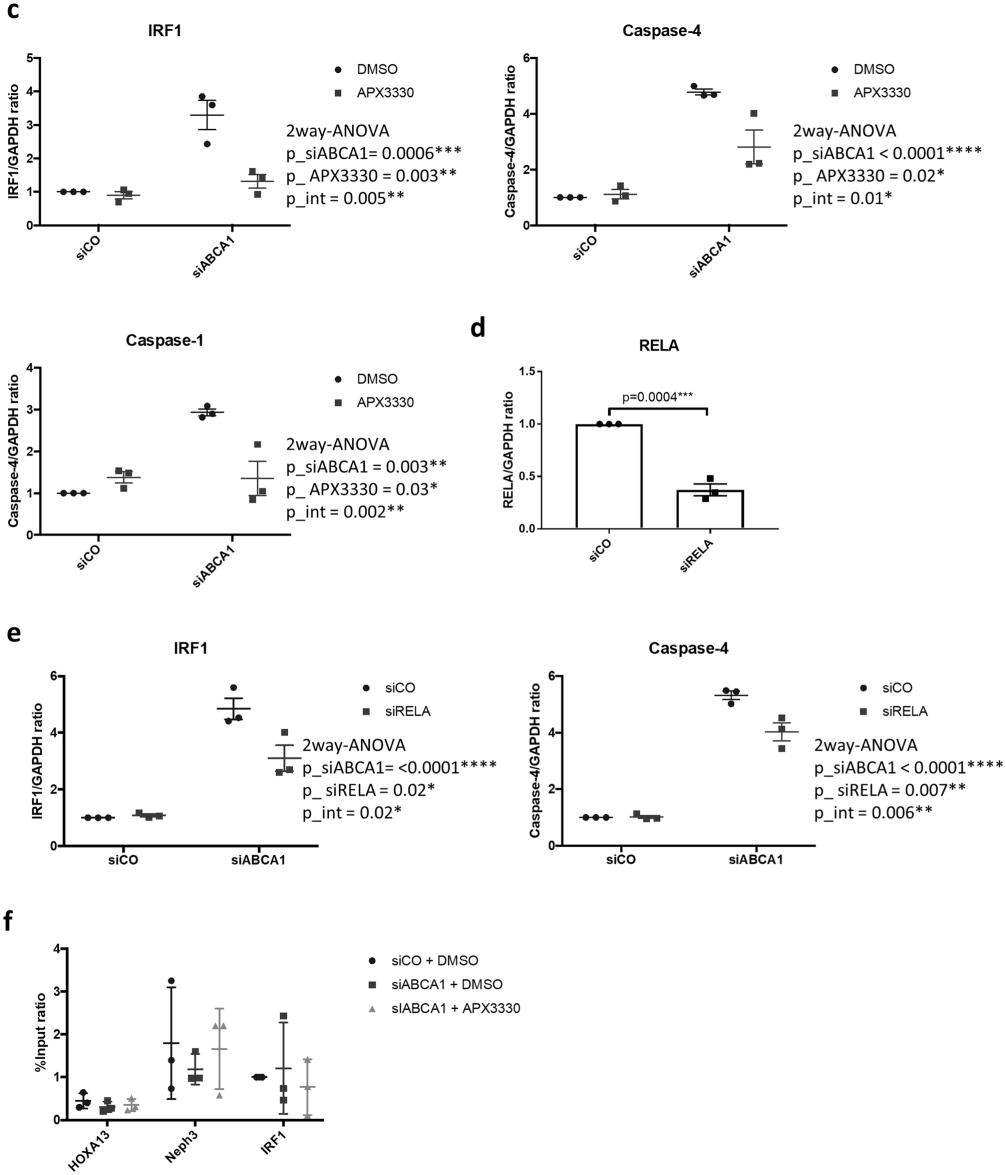
APE1 and NFκB regulates the mRNA expression of IRF1 and caspase-4. (**a**) Protein levels of APE1 in whole cell lysate of siCO and siABCA1 podocytes. The uncropped blots are in the Supplementary Fig. S3a. **(b)** Protein levels of APE1 in nuclear fractions of siCO and siABCA1 podocytes. The uncropped blots are in the Supplementary Fig. S3b. (**c**) mRNA levels of IRF1, caspase-4 and caspase-1 in siCO and siABCA1 podocytes treated with DMSO or APX3330 (APE1 redox inhibitor). (**d**) siRELA knockdown efficiency in siCO podocytes. N = 3 for each group. (**e**) mRNA levels of IRF1 and caspase-4 in siABCA1 podocytes transiently transfected with siCO and siRELA. (**f**) The result of ChIP qPCR for siCO podocytes treated with DMSO, siABCA1 podocytes with DMSO and siABCA1 podocytes with APX3330. Neph3 was used as a positive control and HOXA13 as a negative control for NFκB binding to DNA. N = 3 for each group. *APE1* apurinic/apyrimidinic endonuclease-1, *Neph3* kirre like nephrin family adhesion molecule 2, *HOXA13* Homeobox A13.

In addition to its role in reverse cholesterol transport, ABCA1 also has a role in exporting APE1^7^. APE1, also known as Ref-1 has two functions^20^. First, APE1 reduces transcription factors to activation form and oxidizes itself. Among the transcription factors reduced by APE1 are HIF1α, NFκB, STAT3, AP1 and p53^21^. Second, APE1 works as an apurinic/apyrimidinic endonuclease to nick the abasic site of DNA to prep it for repair. To examine the amount of APE1 in whole cell and nuclei where it functions, we conducted Western blotting for APE1 in whole cell lysate and nuclear fractions of control and siABCA1 podocytes and found that APE1 was increased in siABCA1 podocytes compared to control both in whole cell lysate and nuclear fractions (Fig. 5a,b). Next, we used APX3330, a specific-inhibitor of oxidation–reduction function of APE1, to address the question if the reduction of transcription factors by APE1 contributes to the priming of non-canonical pyroptosis in siABCA1 podocytes. The APE1 redox inhibitor offset the increase of siABCA1-induced mRNA transcription of IRF1 and caspase-4, suggesting a role for APE1 in this process (Fig. 5c).

### NFκB is involved in IRF1 upregulation induced by ABCA1 downregulation

NFκB is a transcription factor that plays an important role in pyroptosis priming^9^. Several signaling pathways activate NFκB, including reactive oxygen species or TLR4 signaling^22–24^. NFκB can directly induce IRF1 transcription in immune cells while IRF1 can indirectly affect the regulation of NFκB^25,26^. To investigate whether NFκB is involved in the upregulation of IRF1 in siABCA1 podocytes, we used siRNA knockdown for RELA, the NFκB p65 subunit, in siCO and siABCA1 podocytes (Fig. 5d). We found a significant statistical interaction with siABCA1 and siRELA in IRF1 and caspase-4 (p_int = 0.02 for IRF1 and 0.006 for caspase-4), which indicated the role of NFκB in siABCA1-induced upregulation of IRF1 and caspase-4 (Fig. 5e). However, we were unable to confirm that the binding of NFκB to binding sites in IRF1 was increased with siABCA1 podocytes (Fig. 5f).

### APE1, IRF1 and caspase-11 are increased in glomeruli of BTBR ob/ob mice

**Figure 6:**
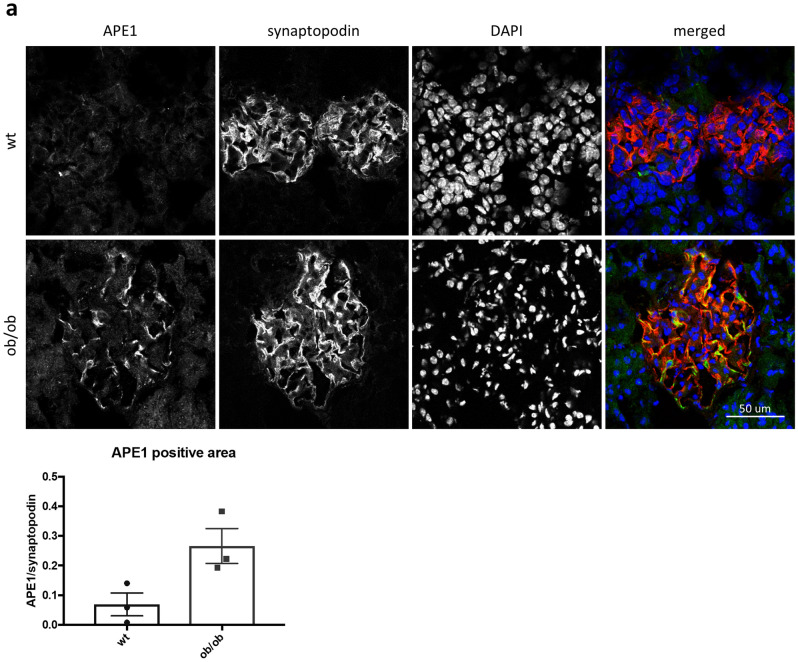

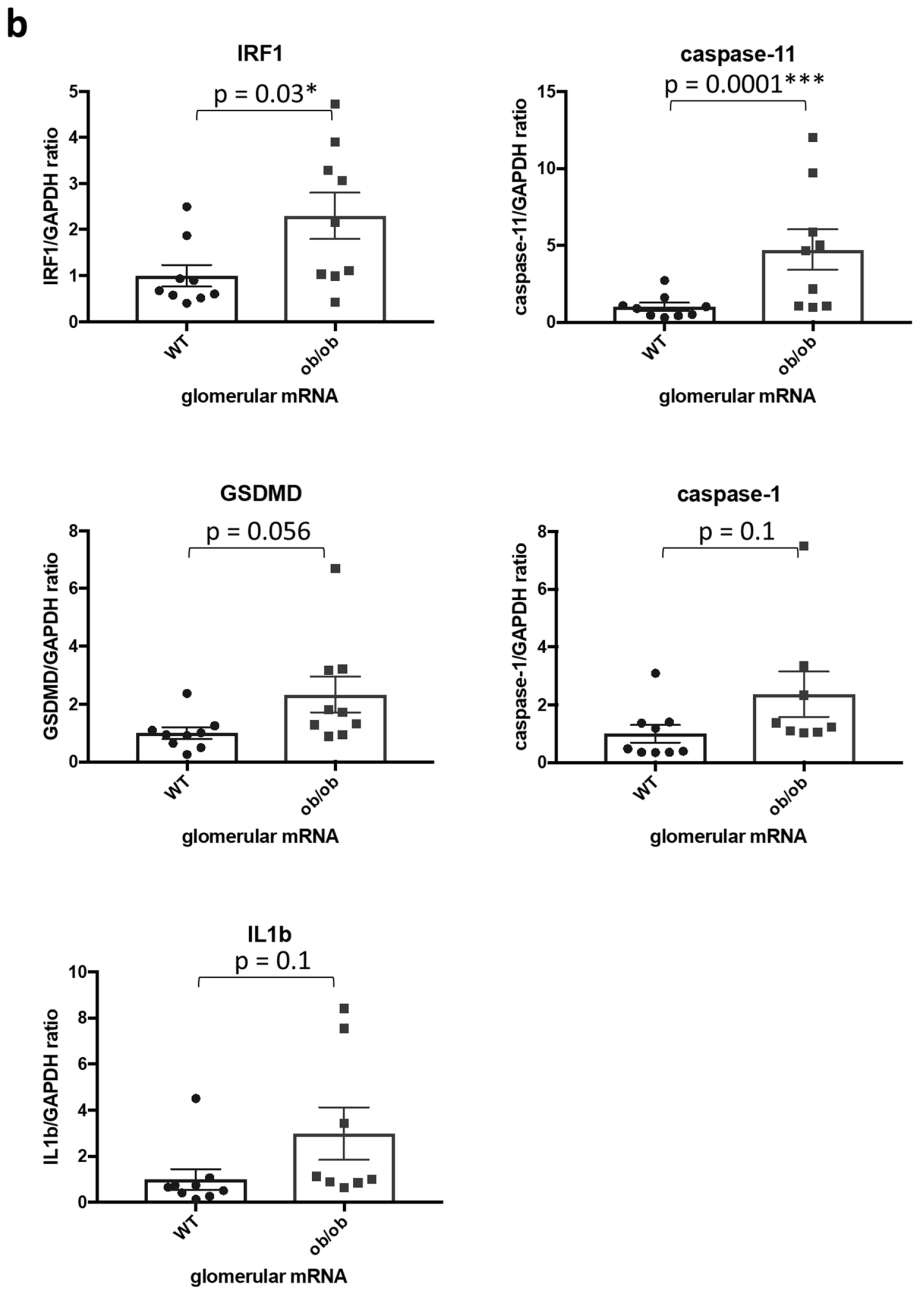
APE1, IRF1 and non-canonical pyroptosis-related genes are increased in glomeruli of ob/ob mice. (**a**) Representative images of immunofluorescence staining for APE1 (green), synaptopodin (red) and nuclei (blue) in kidney tissue sections of wt and BTBR ob/ob mice and average APE1 positive area/synaptopodin positive area of 10 glomeruli in each mouse. N = 3 for each group. (**b**) mRNA levels of IRF1 and non-canonical pyroptosis-related genes in wt and ob/ob mouse glomeruli. Caspase-11 is the mouse ortholog of caspase-4 in human. N = 9 for each group except for caspase-1 and IL1β in ob/ob, which is n = 8.

Finally, to investigate whether our in vitro observations translate in vivo, we used BTBR ob/ob mice, a mouse model of DKD. We previously demonstrated that decreased ABCA1 expression in podocytes plays an important role in DKD progression^5,6^. Immunofluorescence staining demonstrated increased APE1 expression in glomeruli of BTBR ob/ob compared to WT mice (Fig. 6a). In support, we found increased mRNA levels of IRF1 and caspase-11, which is compatible with the activation of the ABCA1/APE1/IRF1 axis in vivo as well as in vitro in DKD (Fig. 6b).

## Discussion

**Figure 7:**
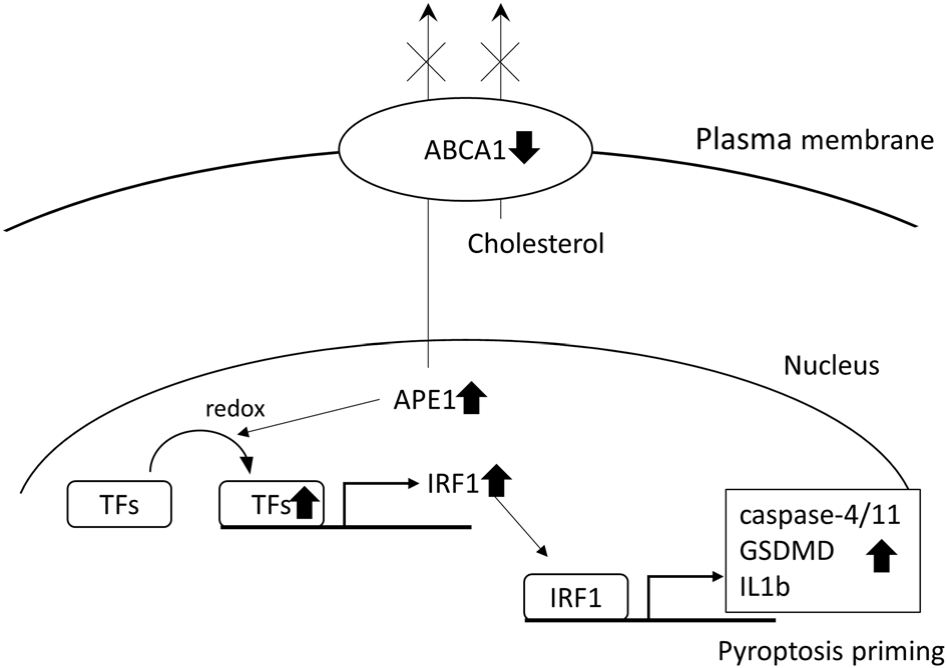
Schematic of the hypothesis. ABCA1 deficiency in DKD causes accumulation of APE1 in nuclei, which activates transcription factors including NFκB via redox reaction. Activated transcription factors (TFs) increases IRF1 expression and IRF1 subsequently increases the expression of non-canonical pyroptosis-related genes such as caspase-4/11, GSDMD and IL1β. TFs: transcription factors.

In this study, we demonstrated that ABCA1 deficiency in podocytes, which we previously showed to contribute to the progression of DKD, induced non-canonical pyroptosis priming in association with nuclear APE1 accumulation and subsequent activation of transcription factors by redox reaction (Fig. 7). Among the non-canonical pyroptosis-related genes upregulated in siABCA1 podocytes, caspase-4/11, GSDMD and IL1β were identified to be regulated by IRF1, while caspase-1 was not regulated by IRF1 but increased by APE1 redox reaction.

Although the function of ABCA1 has been largely attributed to cholesterol efflux, ABCA1 is also involved in other cellular export mechanisms^27^. ABCA1 participates in the secretion of phospholipids such as cardiolipin, lysophosphatidylcholine and phosphatidylserine, extracellular vesicles and proteins associated with anti-inflammatory response and apoptotic cell clearance^28–31^. Recently, a role for ABCA1 in APE1 export has been described^7^.

APE1 mainly has two functions, an endonuclease and a redox function^20,32^. In its redox function, APE1 is oxidized and a transcription factor is reduced through a thiol/sulfide exchange and activated by facilitated DNA binding. Those transcription factors include NFκB, Hypoxia Inducible Factor (HIF), Signal Transducer and Activator of Transcription 3 (STAT3), Activator Protein 1 (AP-1) and cAMP-response element binding protein (CREB). So far, in canonical and non-canonical pyroptosis priming, NFκB was shown to be activated via phosphorylation of inhibitor of NFκB (IκB) kinases by TLR4 signaling^22,33,34^. The canonical pyroptosis pathway involving NLRP3 contributes to DKD but a contribution of non-canonical pyroptosis was also recently reported^11,35^. This study demonstrates that ABCA1 deficiency in podocytes, which we previously demonstrated to play an important role in DKD progression^5,6^, leads to APE1 accumulation and activation of transcription factors by redox reaction of APE1, not by TLR4, in the absence of NLRP3 increase. Thus, this pathway could represent a new mechanism of non-canonical pyroptosis priming in DKD. Our data suggest that APE1 activates several transcription factors and that the combination of their activation could lead to a distinct pattern of gene expression in siABCA1 podocytes. However, further investigation to elucidate the exact details of this process are warranted. It can also be speculated that the involvement of APE1 in mitochondrial function via the regulation of mitochondrial RNA may contribute to DKD^32^.

Priming of pyroptosis in the absence of an activation or cleavage of pyroptosis-related proteins occurred in siABCA1 podocytes, consistent with our previous observation in vitro and in vivo that ABCA1 downregulation itself is not sufficient to cause injury^6^. Nevertheless, ABCA1 induction has been explored as a treatment for DKD^36^. APE1 has been investigated as a treatment target of malignancy, ocular diseases and inflammatory bowel disease^20,32^, and this study indicates the possibility that it could be a target of DKD as well as IRF1.

We have previously published that oxidized cardiolipin is suggested to be implicated in the sensitization of siABCA1 podocytes to DKD.^6^ Some relationship between oxidized phospholipids (oxPLs) and non-canonical pyroptosis are described so far. Oxidized 1-palmitoyl-2-arachidonoyl-*sn*-glycero-3-phosphorylcholine (oxPAPC) can bind caspase-11 and activate it like LPS^37^. On the other hand, although we do not know specifically whether oxidized cardiolipin induces pyroptosis, some other oxPLs are demonstrated to sensitize bone marrow-derived macrophages to N-GSDMD-induced cytotoxicity^38^. It is possible that oxPLs and non-canonical pyroptosis mutually affect each other in ABCA1 downregulated podocytes.

In summary, we demonstrated that ABCA1 deficiency in podocytes, which contributes to DKD progression, induces the expression of non-canonical pyroptosis-related genes such as caspase-4/11, GSDMD, caspase-1 and IL1β via nuclear APE1 accumulation and subsequent reduction of transcription factors. Thus, non-canonical pyroptosis priming by ABCA1/APE1/IRF1 axis could represent a novel pathway to be targeted for the treatment of patients with DKD.

## Materials and methods

### Cell culture

Human podocytes (gift from Dr. Moin Saleem, University of Bristol, Bristol, England) were grown at 33 °C under permissive conditions in RPMI culture medium containing 10% FBS and 1% penicillin/streptomycin and 0.01 mg/ml recombinant human insulin, 0.0055 mg/ml human transferrin (substantially iron-free), and 0.005 μg/ml sodium selenite^39^. Human podocytes were then thermoshifted and differentiated for 14 days at 37 °C in RPMI medium 10% FBS and 1%penicillin/streptomycin. ABCA1 siRNA knockdown (siABCA1) and non-targeting siRNA control (siCO) podocytes were generated and validated as previously described^40^. On day 10–14 of differentiation, podocytes were collected and analyzed. For APE1 inhibition, podocytes were treated with 1 uM TAK-242 (Milipore Sigma, St. Louis MO, USA) for 24 h or 75 uM APX3330 (NOVUS biologicals, Centennial, CO, USA) for 48 h.

### Animals

All animal studies were approved by the Institutional Animal Care and Use Committee (IACUC) at the University of Miami. All experiments were performed in accordance with the guidelines and regulations of IACUC at the University of Miami. Mice were sacrificed at 20 weeks by isoflurane inhalation. The authors complied with the ARRIVE guidelines. All the samples used for this manuscript including mRNA of glomeruli and frozen sections of kidneys of wildtype (wt) and BTBR ob/ob mice were collected and stored for our previous paper^6^ and were newly analyzed. The detailed methods are described previously^6^. 9 mice (5 females and 4 males) each were included in wt and ob/ob group. For immunofluorescence, 3 mice from each group which show typical pathological findings of normal or diabetic kidney disease in paraffin sections were selected and tissue in OCT from those 6 mice were freshly cut and analyzed. For qPCR, all the samples were analyzed, but because of the shortage of one sample, only 8 samples were analyzed for caspase-1 and IL1β in ob/ob group.

### siRNA transfection

We used commercial siRNAs against human IRF1 (Silencer Select, s7501, s7503, Life Technologies Corporation, Carlsbad, CA, USA), RELA (Silencer Select, s11914, Life Technologies Corporation, Carlsbad, CA, USA) and a negative control siRNA (SIC001, Milipore Sigma, St. Louis MO, USA and Silencer Select#1, Life Technologies Corporation, Carlsbad, CA, USA). These siRNAs were introduced into podocytes using HiPerfect (QIAGEN, Hilden, Germany) according to the manufacturer’s instructions.

### Real-time quantitative PCR (qPCR)

Cell RNA was isolated with RNeasy Plus Mini Kit (QIAGEN, Hilden, Germany) and reverse transcribed using qScript DNA SuperMix (Quantabio, Beverly, MA, USA). PCR was performed on a StepOnePlus system (Applied biosystems, Waltham, MA, USA) with PerfeCTa SYBER Green SuperMix (Quantabio, Beverly, MA, USA). The data were normalized to GAPDH. Primer sequences are listed in Table 1. We repeated at least 3 transient transfections in siRNA KD. Each point per group represents a different transfection of cells. For IL1b mRNA expression and IRF2 mRNA expression we repeated the experiment 4 or 6 times respectively to reach statistical significance because of wide standard deviation.

**Table 1:** Primers used in the article.

Gene	Use	Species		Sequence
*Caspase-4*	RT-PCR	Human	Forward	AGATGCCCTCAAGCTTTGTC
			Reverse	TGCGGTTGTTTCTCTCCTTT
*GSDMD*	RT-PCR	Human	Forward	GCCTCCACAACTTCCTGACAGATG
			Reverse	GGTCTCCACCTCTGCCCGTAG
*Caspase-1*	RT-PCR	Human	Forward	CACACCGCCCAGAGCACAAG
			Reverse	TCCCACAAATGCCTTCCCGAATAC
*IL1b*	RT-PCR	Human	Forward	TTCGACACATGGGATAACGAGG
			Reverse	TTTTTGCTGTGAGTCCCGGAG
*NLRP3*	RT-PCR	Human	Forward	CTTCTCTGATGAGGCCCAAG
			Reverse	GCAGCAAACTGGAAAGGAAG
*IRF1*	RT-PCR	Human	Forward	CCAGAGCAGGAACAAGGG
			Reverse	GGTCATCAGGCAGAGTGGA
*IRF2*	RT-PCR	Human	Forward	TGGATGCATGCGGCTAGA
			Reverse	CATCTGAAATTCGCCTTCC
*GAPDH*	RT-PCR	Human	Forward	TGCACCACCAACTGCTTAGC
			Reverse	GGCATGGACTGTGGTCATGAG
*Caspase-11*	RT-PCR	Mouse	Forward	CCTGAAGAGTTCACAAGGCTT
			Reverse	CCTTTCGTGTACGGCCATTG
*GSDMD*	RT-PCR	Mouse	Forward	CCATCGGCCTTTGAGAAAGTG
			Reverse	ACACATGAATAACGGGGTTTCC
*Caspase-1*	RT-PCR	Mouse	Forward	AATGAAGTTGCTGCTGGAGGA
			Reverse	CAGAAGTCTTGTGCTCTGGGC
*IL1b*	RT-PCR	Mouse	Forward	GCAACTGTTCCTGAACTCAACT
			Reverse	ATCTTTTGGGGTCCGTCAACT
*IRF1*	RT-PCR	Mouse	Forward	CTTCGTCGAGGTAGGACGTG
			Reverse	CTTTGCTGCAGGAGCGATTC
*GAPDH*	RT-PCR	Mouse	Forward	GAAGGGCTCATGACCACA
			Reverse	GGATGCAGGGATGATGTTCT
*HOXA13*	ChIP	Human	Forward	GCTTCTTTCTCCCCCTCCTA
			Reverse	CCGATCCCTGTGTAACTTGC
*Neph3*	ChIP	Human	Forward	GAGTTTCTCAACGGGAAGAG
			Reverse	GCCTCTGACGCTCTGAAAC
*IRF1*	ChIP	Human	Forward	CCACCGAGCAATCCAAACAC
			Reverse	GCCTGATTTCCCCGAAATGAC

Sequences of the primers used for RT-PCR and ChIP in the article.

### Western blotting

Cells lysates were prepared using RIPA buffer. Protein concentration was measured with the bicinchoninic acid (BCA) reagent (Thermo Fisher Scientific Inc., Waltham, MA, USA). 20–30 μg of protein extract was loaded onto 4 to 20% SDS–polyacrylamide gel electrophoresis (SDS-PAGE) gels (Bio-Rad Laboratories Inc., Hercules, A, USA) and transferred to Immobilon-P PVDF membranes (Bio-Rad Laboratories Inc., Hercules, A, USA). Western blot analysis was performed using following primary antibodies: caspase-4 ($4450, rabbit, 1:1000), caspase-1 (#3866, rabbit, 1:1000 all from Cell Signaling Technology Inc.), IL1β (AF-201-NA, goat, 1:1000, R and D systems), GSDMD (ab210070, rabbit, 1:1000, Abcam PLC), N-GSDMD (ab215203, rabbit, 1:1000, Abcam PLC), APE1 (NB100-116, mouse, 1:1000, NOVUS biologicals), GAPDH (CB1001, mouse, 1:10,000, Sigma-Aldrich), beta actin (A3854, mouse, 1:10,000, Sigma-Aldrich); or secondary antibodies: anti-mouse IgG HRP (Promega, W402B, 1:10,000), anti-rabbit IgG HRP (Promega, W401B, 1:10,000), anti-goat IgG HRP (Promega, V8051, 1:10,000). Signal was detected with Radiance ECL (Azure Biosystems Inc., Dublin, CA, USA) or WesternBright ECL (Advansta Inc., San Jose, CA, USA) using Azure c600 Imaging System.

Protein from supernatants were isolated using Spin-X UF Concentrator (Corning Inc., Corning, NY, USA) and the values were normalized to the protein concentration of cells in the medium.

Nuclear fractions were obtained using a Cell Fractionation Kit (ab109719, Abcam PLC, Cambridge, UK) according to the manufacturer’s instructions.

### LPS electroporation

LPS wea electroporated into podocytes using program T-020 in Nucleofector 2b (Lonza, Valais, Switzerland) and Amexa Basic Nucleofector Kit for Primary Mammalian epithelial cells (VPI-1005) according to manufacturer’s instruction. LPS-EB ultrapure (Invivogen, San Diego, CA, USA) was added to differentiated podocytes at a concentration of 3.0 ng/400,000 cells. The supernatants were collected at 4 h after electroporation and protein from them were collected using Amicon Ultra (Merck, Darmstadt, Germany) and analyzed with western blotting. Samples were normalized to mL/number of cells in the medium. Z-VAD-fmk (Selleck Biotech, Tokyo, Japan) was added 1 h prior to, during and after electroporation at a concentration of 20 nM.

### Chromatin Immunoprecipitation (ChIP)

The binding of NFκB to the IRF1 promoter was assessed using the chromatin immunoprecipitation assay. Podocytes were cross-linked for 10 min using 1% paraformaldehyde and sonicated into fragments. The fragmented DNA forming a complex with NFκB was immunoprecipitated with an anti- NFκB rabbit polyclonal antibody (Cell Signaling Technology, Inc. Danvers, MA, USA) and Dynabeads M-280 Sheep Anti-Rabbit IgG (Invitrogen, St. Louis MO, USA). The precipitated DNA was used as a template for PCR reactions with the primers listed in Table 1. Five NFκB binding sites in IRF promoter region (− 500 to + 1500 from the transcription starting point) were predicted by FIMO database and the one nearest to the transcription starting site was selected, which was consistent with the publicly available ChIP-seq data which showed a strong peak near the transcription starting site in B-lymphocytes (GSM935478, GSM935526, GSM935285, GSM5529, GSM935531, GSM935273 and GSM935279)^25^. Neph3 (also known as kirre like nephrin family adhesion molecule 2) promoter region has known NFκB-binding site, and was used as a positive control^41^. Homeobox A13 (HOXA13) is the negative-control primer, which was designed for the promoter regions of chromosome 20.

### Immunofluorescence

For immunofluorescence staining of kidneys from mice, fresh cut 4 μm-thick tissue in OCT was used. No fixation agent was applied. Permeabilization was performed using 0.3% Triton X-100 in 1 × PBS for 15 min at room temperature. A blocking step was performed using Power Block Universal Blocking Reagent (BioGenex, Fremont CA, USA) for 10 min at room temperature. Primary antibodies (for APE1 (NB100-101, rabbit, 1:500, NOVUS biologicals) and synaptopodin (sc-21537, goat, 1:500, Santa Cruz Biotechnolgy Inc.) were used for 24 h at 4 °C. Secondary antibodies (anti-rabbit Alexa Flour 488 (A32790, donkey, Invitrogen) and anti-goat Alexa Flour 568 (A11057, donkey, Invitrogen)) were used for 1 h at room temperature. The anti-caspase-11 antibody was conjugated with Alexa Flour 594 and used without secondary antibodies. To detect nucleus, ProLong Gold antifade reagent with DAPI (Invitrogen, St. Louis MO, USA) was applied.

Images were acquired by laser scanning confocal microscopy using a Leica SP5 Inverted microscope, × 20 objective (Leica Microsystems CMS GmbH, Germany). Measuring of cell fluorescence was performed using Image J software.

### Statistical analysis

All the data are reported as mean ± standard error of mean and as individual values in the dot plots. Two groups were compared using independent sample t-test and a two-way ANOVA test was used to analyze the effect of two independent factors on outcomes and investigate if there is an interaction effect between the two factors on the outcomes. A p-value < 0.05 was considered statistically significant for all tests. GraphPad Prism, version 7.0 (GraphPad Software Inc., San Diego, CA, USA) was used for data analyses.

## Supplementary Information

Supplementary Figures.

## Data Availability

The data underlying this article are available in the article. The datasets referred to in the current study are available in the the Gene Expression Omnibus (GEO) repository, GSM935478, GSM935526, GSM935285, GSM5529, GSM935531, GSM935273 and GSM935279.
