# Supplementary material for: ABCA1 deficiency contributes to podocyte pyroptosis priming via the APE1/IRF1 axis in diabetic kidney disease
Source: Sci Rep. 2023 Jun 14;13:9616. doi: 10.1038/s41598-023-35499-5 (PMC10267156; doi:10.1038/s41598-023-35499-5)
Supplement: Supplementary file 1 — Supplementary Figures. [file 41598_2023_35499_MOESM1_ESM.pptx]

## Slide 1
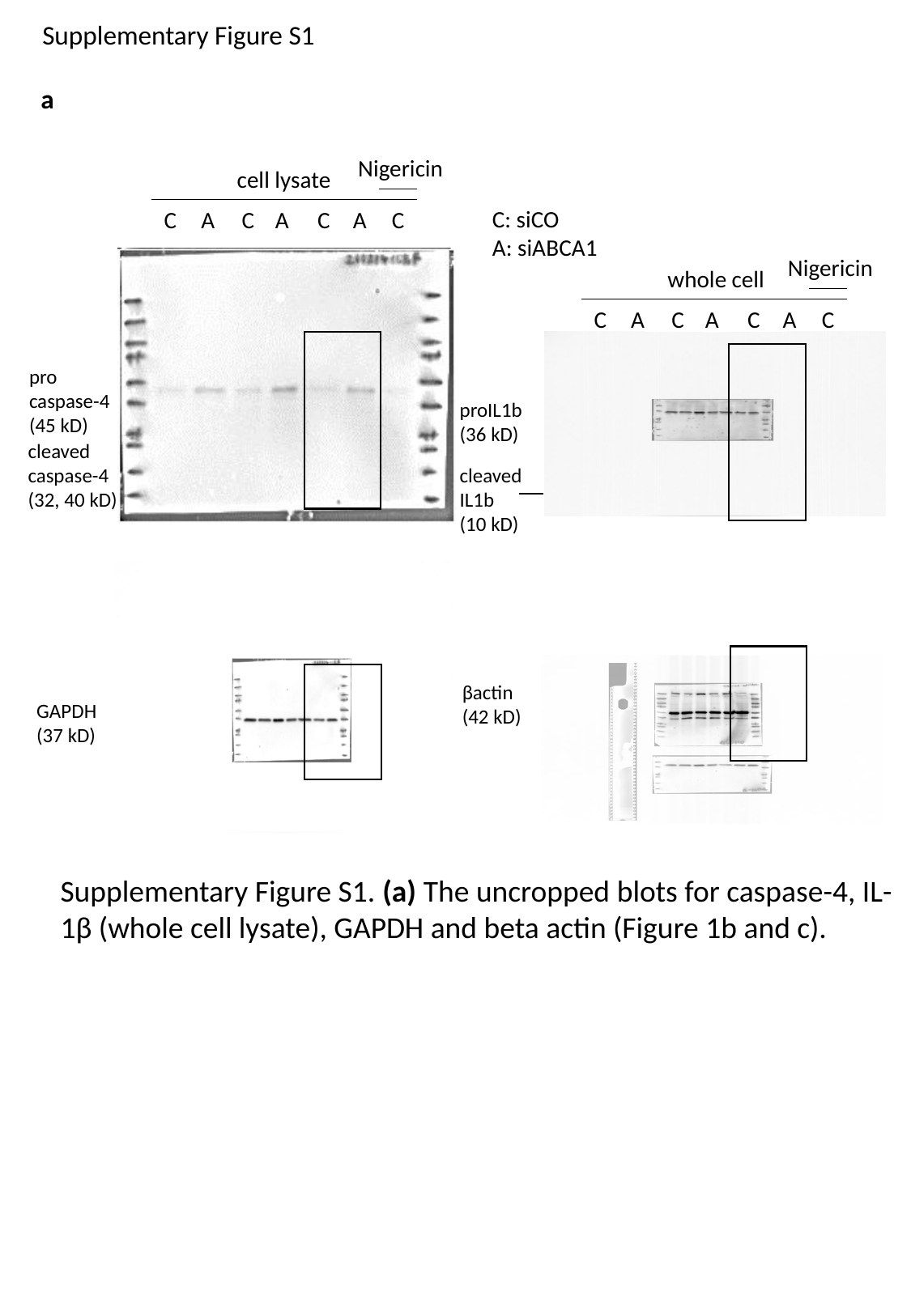

Supplementary Figure S1
a
Nigericin
cell lysate
C
A
C
A
C
A
C
C: siCO
A: siABCA1
Nigericin
whole cell
C
A
C
A
C
A
C
pro
caspase-4
(45 kD)
proIL1b
(36 kD)
cleaved
caspase-4
(32, 40 kD)
cleaved IL1b
(10 kD)
βactin
(42 kD)
GAPDH
(37 kD)
Supplementary Figure S1. (a) The uncropped blots for caspase-4, IL-1β (whole cell lysate), GAPDH and beta actin (Figure 1b and c).

## Slide 2
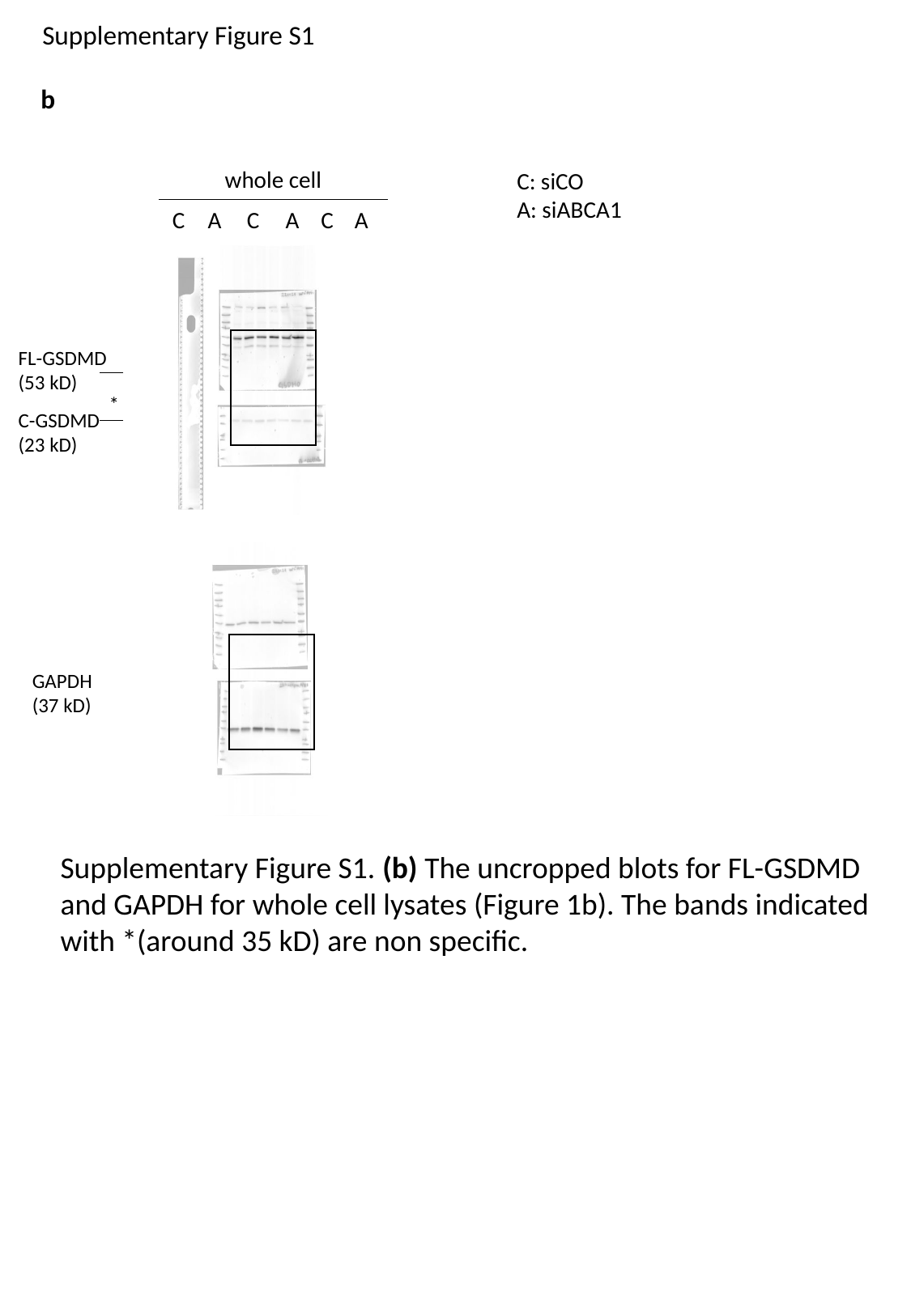

Supplementary Figure S1
b
whole cell
C
A
C
A
C
A
C: siCO
A: siABCA1
FL-GSDMD
(53 kD)
*
C-GSDMD
(23 kD)
GAPDH
(37 kD)
Supplementary Figure S1. (b) The uncropped blots for FL-GSDMD and GAPDH for whole cell lysates (Figure 1b). The bands indicated with *(around 35 kD) are non specific.

## Slide 3
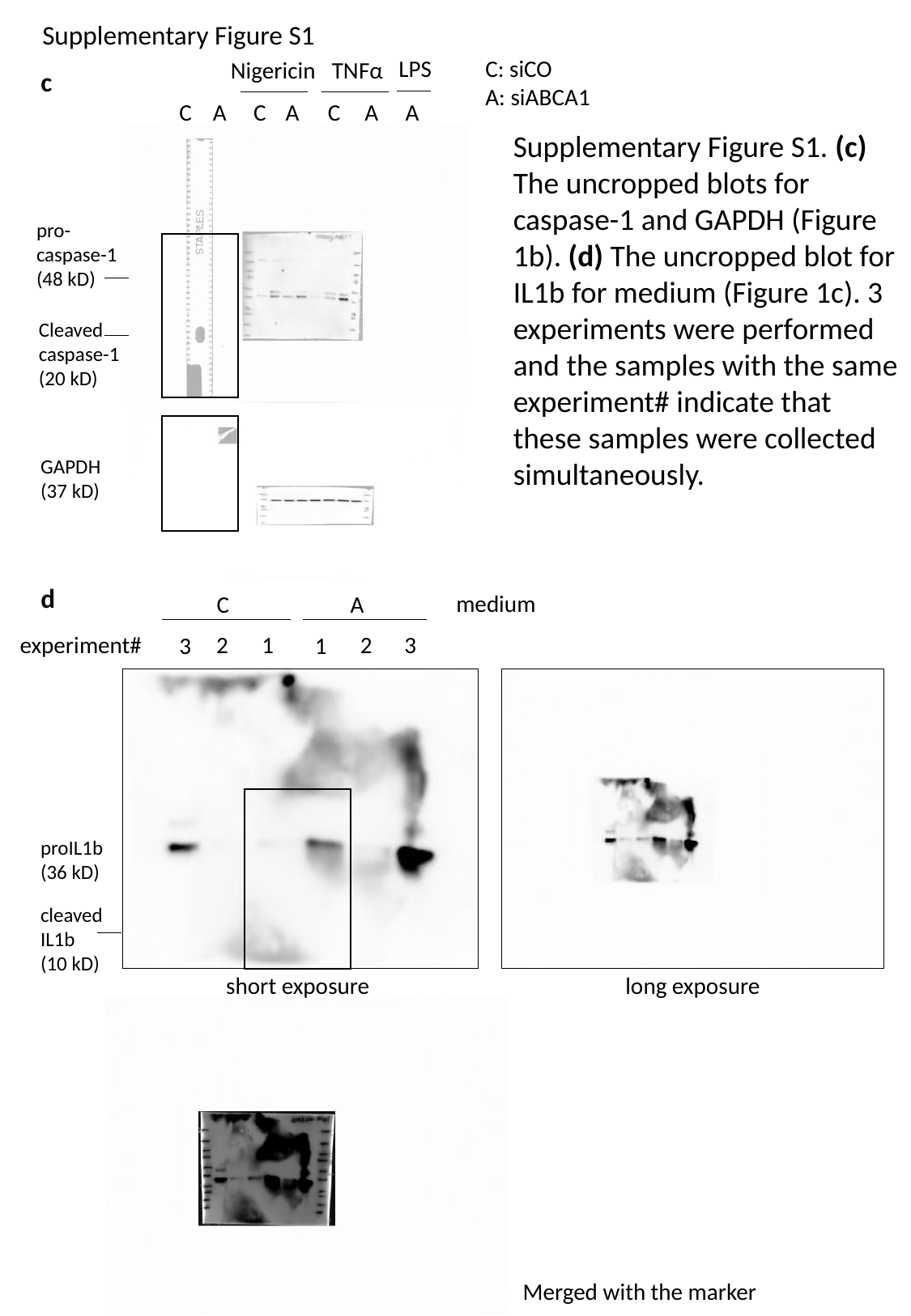

Supplementary Figure S1
LPS
C: siCO
A: siABCA1
TNFα
Nigericin
C
A
C
A
C
A
A
c
Supplementary Figure S1. (c) The uncropped blots for caspase-1 and GAPDH (Figure 1b). (d) The uncropped blot for IL1b for medium (Figure 1c). 3 experiments were performed and the samples with the same experiment# indicate that these samples were collected simultaneously.
pro-caspase-1
(48 kD)
Cleaved
caspase-1
(20 kD)
GAPDH
(37 kD)
d
medium
C
A
experiment#
2
1
2
3
3
1
proIL1b
(36 kD)
cleaved
IL1b
(10 kD)
short exposure
long exposure
Merged with the marker

## Slide 4
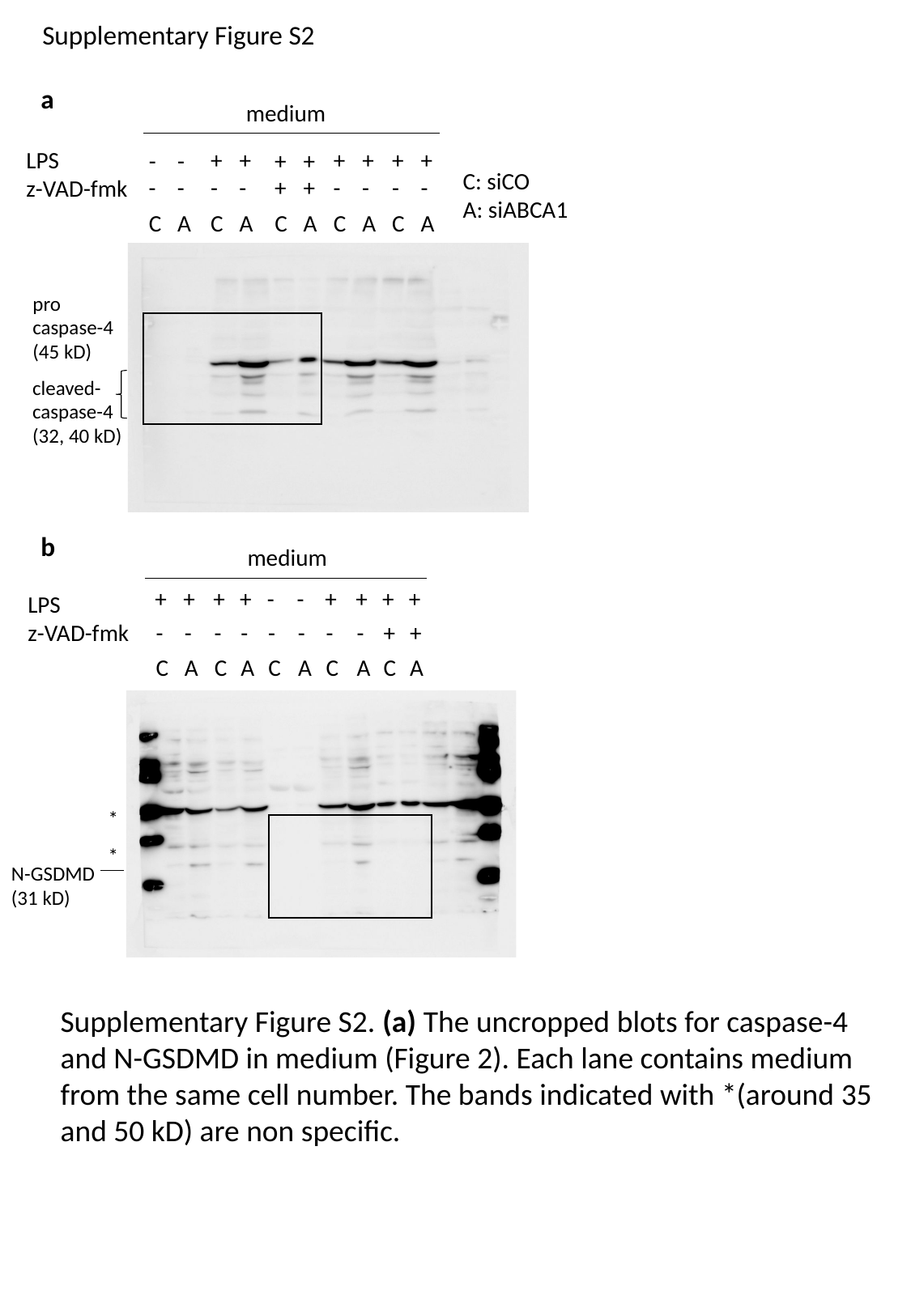

Supplementary Figure S2
a
medium
LPS
z-VAD-fmk
+
+
+
+
+
+
+
+
-
-
-
-
-
-
-
-
+
+
-
-
C
A
C
A
C
A
C
A
C
A
C: siCO
A: siABCA1
pro
caspase-4
(45 kD)
cleaved-
caspase-4
(32, 40 kD)
b
medium
+
+
+
+
+
+
-
-
+
+
LPS
z-VAD-fmk
-
-
-
-
+
+
-
-
-
-
C
A
C
A
C
A
C
A
C
A
*
*
N-GSDMD
(31 kD)
Supplementary Figure S2. (a) The uncropped blots for caspase-4 and N-GSDMD in medium (Figure 2). Each lane contains medium from the same cell number. The bands indicated with *(around 35 and 50 kD) are non specific.

## Slide 5
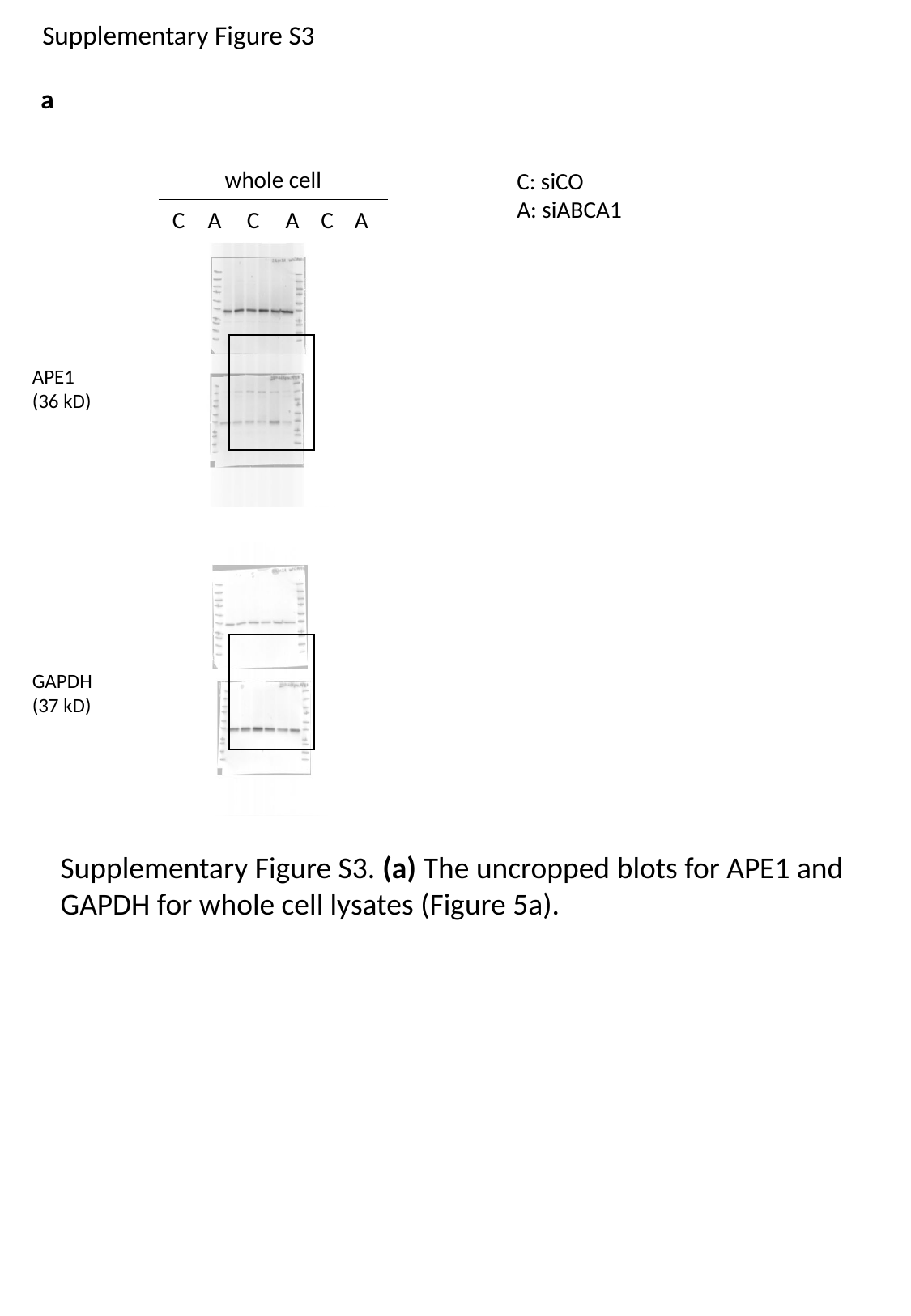

Supplementary Figure S3
a
whole cell
C
A
C
A
C
A
C: siCO
A: siABCA1
APE1
(36 kD)
GAPDH
(37 kD)
Supplementary Figure S3. (a) The uncropped blots for APE1 and GAPDH for whole cell lysates (Figure 5a).

## Slide 6
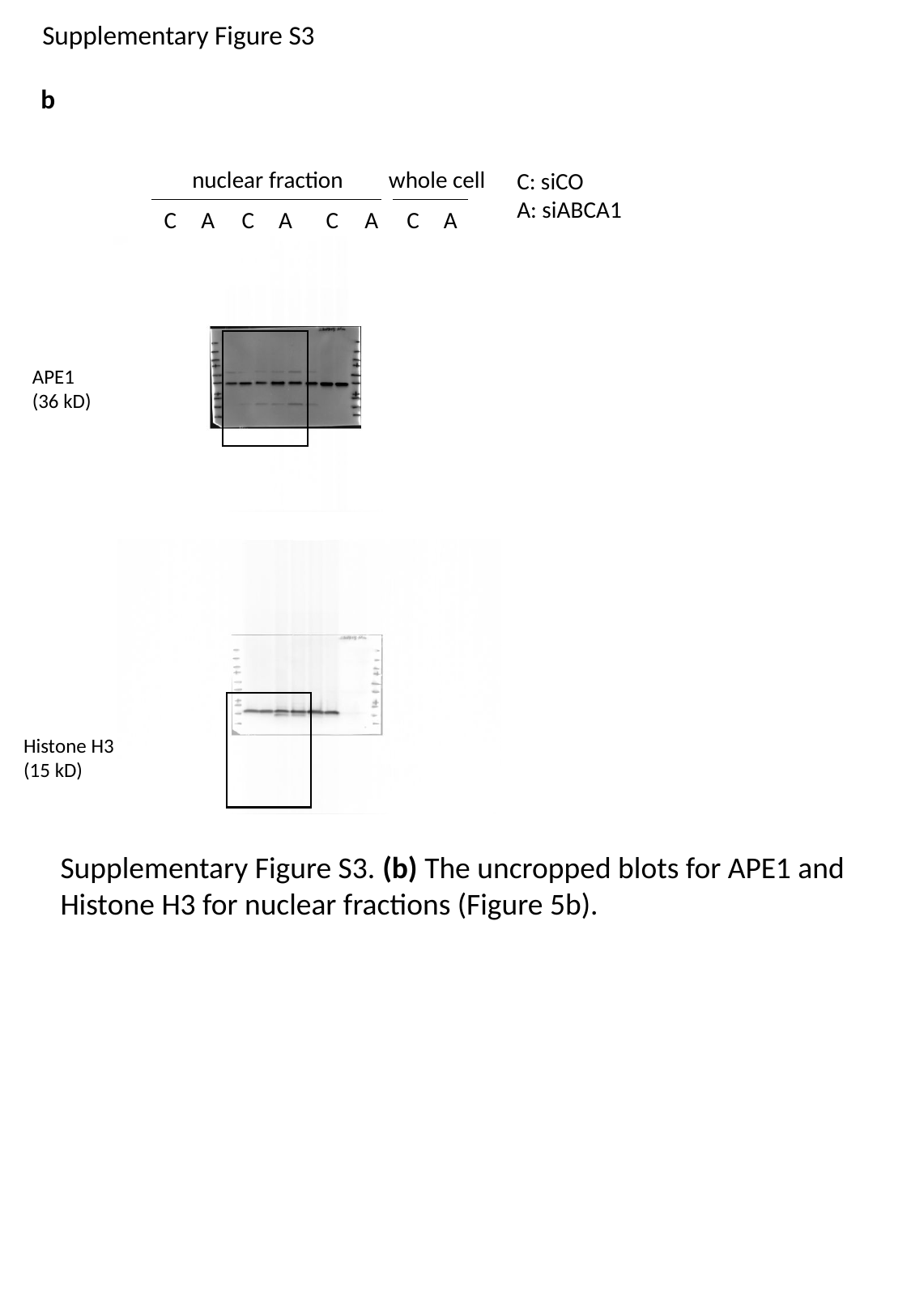

Supplementary Figure S3
b
whole cell
nuclear fraction
C
A
C
A
C
A
C: siCO
A: siABCA1
C
A
APE1
(36 kD)
Histone H3
(15 kD)
Supplementary Figure S3. (b) The uncropped blots for APE1 and Histone H3 for nuclear fractions (Figure 5b).

## Slide 7
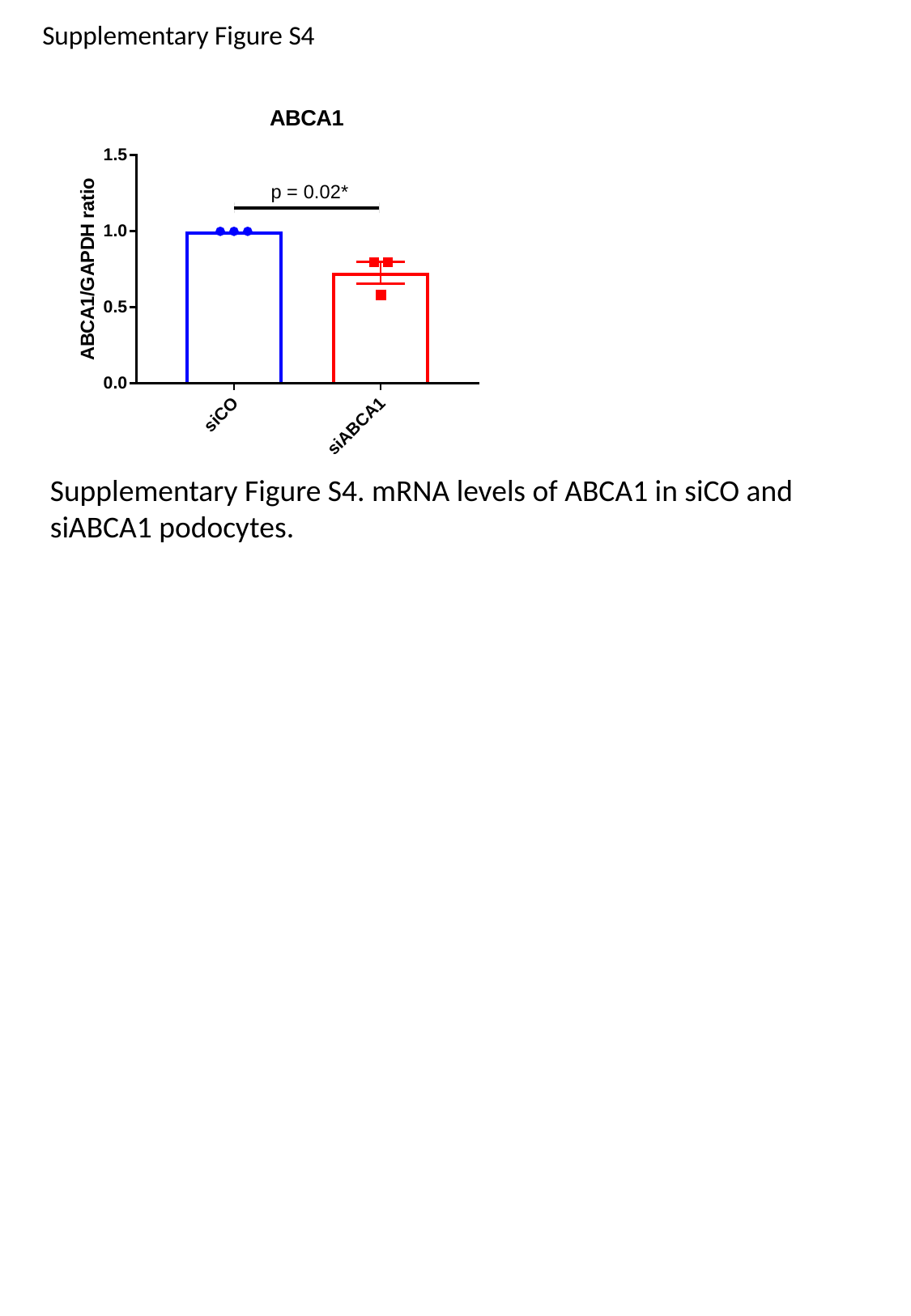

Supplementary Figure S4
Supplementary Figure S4. mRNA levels of ABCA1 in siCO and siABCA1 podocytes.
